# Stockton's Dental Intelligencer

**Published:** 1845-09

**Authors:** 


### *Stockton's Dental Intelligencer.*

This paper contains many very valuable and highly interesting articles on the science and art of dental surgery; and the uncompromising stand it has taken against empiricism, has enabled it to exert a wide and salutary influence. When it was first started, it was thought by many that it would be discontinued after the issue of three or four, or half a dozen numbers; but, true to his promise, the publisher has now completed the first volume, and, as we learn, is about to commence the second. We wish him success in his undertaking; and the cheapness of the publication, for the amount of valuable reading matter which it contains, commends itself to the patronage of the profession generally.

All efforts to diffuse correct information on this important branch of the healing art should be encouraged. It is due to the public that every practitioner should avail himself of every means of professional information within his reach, and that he labors diligently and constantly to perfect himself in the knowledge of his pursuit. Experience, observation, and research are now daily erecting higher, and still higher standards of skill in this, as well as in all the other departments of medicine, so that a much greater amount of industry and effort are required to keep up with the improvements of the present day than were necessary to keep pace with the progress of the art, half or even a quarter of a century ago; and he who has not ambition and pride of character enough to use sufficient industry, and exert the necessary effort to acquaint himself with them, should at once quit the pursuit, and engage in something which requires a less vigorous exercise of his intellectual and physical energies. The practitioner of dental surgery, no matter what may be his qualifications, should labor hard, and constantly, for higher professional attainments, and never rest satisfied with his present acquirements.

